# Fairness and Risk: An Ethical Argument for a Group Fairness Definition Insurers Can Use

**DOI:** 10.1007/s13347-023-00624-9

**Published:** 2023-06-19

**Authors:** Joachim Baumann, Michele Loi

**Affiliations:** 1 Department of Informatics, University of Zurich, Zurich, Switzerland; 2 School of Engineering, Zurich University of Applied Sciences, Zurich, Switzerland; 3 Department of Mathematics, Politecnico Di Milano, Milan, Italy

**Keywords:** Algorithmic fairness, Risk, Moral philosophy, Actuarial fairness, Group fairness criteria, Sufficiency, Prediction-based decision making

## Abstract

Algorithmic predictions are promising for insurance companies to develop personalized risk models for determining premiums. In this context, issues of fairness, discrimination, and social injustice might arise: Algorithms for estimating the risk based on personal data may be biased towards specific social groups, leading to systematic disadvantages for those groups. Personalized premiums may thus lead to discrimination and social injustice. It is well known from many application fields that such biases occur frequently and naturally when prediction models are applied to people unless special efforts are made to avoid them. Insurance is no exception. In this paper, we provide a thorough analysis of algorithmic fairness in the case of insurance premiums. We ask what “fairness” might mean in this context and how the fairness of a premium system can be measured. For this, we apply the established fairness frameworks of the fair machine learning literature to the case of insurance premiums and show which of the existing fairness criteria can be applied to assess the fairness of insurance premiums. We argue that two of the often-discussed group fairness criteria, *independence* (also called *statistical parity* or *demographic parity*) and *separation* (also known as *equalized odds*), are not normatively appropriate for insurance premiums. Instead, we propose the *sufficiency* criterion (also known as *well-calibration*) as a morally defensible alternative that allows us to test for systematic biases in premiums towards certain groups based on the risk they bring to the pool. In addition, we clarify the connection between group fairness and different degrees of personalization. Our findings enable insurers to assess the fairness properties of their risk models, helping them avoid reputation damage resulting from potentially unfair and discriminatory premium systems.

## Introduction

Insurance companies are believed to rely more and more on algorithmic predictions to develop personalized risk models to determine premiums in the future (Cevolini and Esposito, 2020; Wuthrich, 2020; Wüthrich and Merz, 2023). From the machine learning (ML) literature, we know that issues of fairness, discrimination, and social injustice might arise in this context (Kleinberg et al., 2016; Chouldechova, 2017): Algorithms for estimating the risk based on personal data may be biased towards specific social groups, leading to systematic disadvantages for those groups. To assess the fairness of such systems, many different so-called *fairness criteria* have been proposed (Barocas et al., 2019; Kearns and Roth, 2019; Verma & Rubin, 2018). However, for insurance premiums that are set based on the outcome of personalized risk models, it remains unclear which fairness criteria are relevant. Moreover, the criteria are mathematically incompatible, so they cannot be fulfilled simultaneously (Kleinberg et al., 2016; Chouldechova, 2017; Garg et al., 2020; Friedler et al., 2021). For this reason, there is the need to choose one of the criteria over the others. In this paper, we do this by drawing on fair ML literature and moral philosophy.

Most group fairness criteria fall into one of three categories: *independence*, *separation*, or *sufficiency* (Barocas et al., 2019). Notice that other approaches to fair ML exist, which we do not consider here, such as *individual fairness* (Dwork et al., 2012) or *counterfactual fairness* (Kusner et al., 2017). Further, notice that there is no consensus regarding the terminology: different terms have been used to refer to *independence*, *separation*, and *sufficiency*.^1^ (Baumann et al., 2022).

There is no consensus as to which statistical criterion of fairness is relevant in all cases – indeed, different contexts may call for different criteria. In this paper, we argue for a specific statistical criterion of fairness for private insurers which implement chance solidarity, and we assume that there is no need for income or risk solidarity.^2^

This is a novel and important contribution to the literature for three reasons: First, the debate on algorithmic fairness, in particular in its most theoretical and philosophical variants where simpler examples are preferred, typically considers binary classification problems. For example, a prediction is used to decide whether to award parole to a prisoner. Here there are only two possible outcomes: the prisoner is either released or kept in jail. And there are only two possible justifications for either decision: the prisoner will either reoffend or not. In the insurance case, we deal with a case in which both the decision outcome (how much a client should pay) and the attribute providing a possible justification for the decision (the risk of the client) are non-binary,^3^

Second, few papers combine the ethical and mathematical side of insurance premiums into an organic argument for a specific statistical criterion of fairness.^4^ To our knowledge, Dolman & Semenovich (2018) are the first that attempt to combine group fairness criteria from the fair ML literature with insurance premiums. In particular, they link these group fairness criteria with *actuarial fairness*. However, a normative argument for or against any of the discussed criteria is missing entirely. For the insurance context, such debate cannot disregard the ethical debate that mainly focuses on one often-discussed notion of fairness in the insurance context called *actuarial fairness* (see Section 3.2 for a formal definition). One approach, which favors *actuarial fairness*, is to assess the fairness of insurance premiums by using the actuarial rates as a reference (Miller, 2009). Another approach, put forward by the Council of the European Union (2004), opposes the notion of *actuarial fairness* and instead strives for *equal treatment* despite potentially different risks.

A clear difference between those two competing notions of insurance fairness is that the former tries to estimate the risk as accurately as possible by relying on all available data. In contrast, the latter (at least to some degree) disregards statistical considerations in support of some kind of solidarity. In addition, another strand of the debate on the ethics of insurance premiums simply focuses on whether a certain variable should be used for training.^5^^,^^6^ The degree of personalization indeed has an immediate effect on the algorithmic model as it determines the set of predictor variables (i.e., the feature space) that can be used as training data. However, it is not valid to assume that a certain degree of personalization directly implies a specific level of fairness. The European Court of Justice banned gender-related variables for risk-rating practices in private insurance pricing in 2012^7^ This forced insurance companies to reduce the degree of personalization by excluding *sex* as an explanatory variable for the model training, which, in fact, resulted in higher premiums for everyone (Schanze, 2013). This is in line with Lipton et al. (2018), who found that not using sensitive attributes^8^ is suboptimal with regard to the balance between accuracy and impact parity. Hence, the connection between the chosen degree of personalization and the fairness of the resulting insurance premiums remains unclear.

A recent paper by Hedden (2021) appears to provide an answer in favor of one specific criterion of fairness, *sufficiency*,^9^ and against the other two we discuss here, *separation* and *independence*. However, the thesis of this paper does not derive logically from Hedden’s thesis because Hedden’s argument does not provide any positive argument in favor of *sufficiency* being a criterion of fairness anywhere except by exclusion.^10^ The *conjecture* that *sufficiency* is necessary for fairness is supported by our argument since we argue that it is the relevant criterion for insurance. However, we only argue for a contextual criterion of fairness. So, our argument (just like Hedden’s original one) is also compatible with no statistical fairness measure being necessary for fairness in all cases.^11^

Third, our contributions and findings are both theoretically and practically relevant. We bridge the gap between group fairness criteria (Barocas et al., 2019) and *actuarial fairness*, which is an often-discussed principle of fairness in the insurance context. We show that two of the main group fairness criteria (*independence* and *separation*) are not appropriate for the specific type of insurance premiums we focus on, both on moral grounds and mathematical ones. However, we find that another fairness criterion (*sufficiency*) is both morally defensible and verifiable. In addition, we clarify the connection between group fairness and different degrees of personalization, also illustrated through a practical example. Thus, our results enable insurers to assess the fairness properties of their risk models in relation to the most discussed group fairness criteria from the ML literature. This is achieved by providing a moral justification for selecting the one that is appropriate for the context in which chance solidarity – and no other form of solidarity – is meant to be achieved.

## Approach

In this work, we investigate the fairness of personalized insurance premiums. Just as with any other predictive model that is used for making consequential decisions, the outcomes of personalized risk models used by insurers might be biased towards a specific group, leading to discrimination. As described in the previous section, there are different options to tackle this problem. Any appropriate approach to ensure the fairness of an algorithmic decision making system very much depends on the context it is utilized in. Hence, a normative evaluation specific to the situation in question is necessary before technically implementing a certain fairness-enhancing solution. This evaluation depends on many details, such as how an algorithmic outcome is used to make a decision, the type of decision made, the individuals affected, the algorithm used, and even the data available to train this algorithm. An interdisciplinary approach is needed to account for the desired impacts from an ethical perspective and the technical possibilities to measure the fairness of such a complex decision making system. In this paper, we provide an ethical argument for an appropriate group fairness definition in the context of insurance premiums by following such an interdisciplinary approach. We assume that there is a population that is split into various socio-demographic groups, for example, gender, nationality, ethnic group, or in some contexts, race, age, etc. For ease of exposition, in our examples, we shall use men and women as a simplification of gender. We further assume that there is a risk for every person, defined as the magnitude of a harmful event (e.g., being liable for $1,000 damages after a car collision) times the probability of its occurrence, and there is an insurer that offers insurance against this risk.

A notion of fairness that is often discussed in the context of insurance is *actuarial fairness* (requiring premiums to be set proportional to an individual’s risk). However, because risk cannot be measured at an individual level, this notion of fairness is not appropriate to measure the fairness of insurance premiums (as we will show in Section 3.2). For this reason, we advocate a shift from fairness for individuals to fairness for groups. To do that, we must move from *actuarial fairness* to group fairness notions as mathematical and moral notions of fairness. Since the different fairness criteria are mathematically incompatible (Kleinberg et al., 2016; Chouldechova, 2017; Barocas et al., 2019), the question of which of those should be chosen still remains.^12^

The context of insurance companies who make use of risk prediction models for personalized pricing is conceptually different from the algorithmic decision making systems that the literature on algorithmic fairness usually focuses on. Most papers in this field investigate the fairness of classification algorithms. Some analyze the fairness of regression models. However, they target settings where the decision-relevant attribute is perfectly observable, if only in hindsight. This is not the case for personalized insurance premiums, where prediction models are used to estimate the risk of individuals, which cannot be measured on an individual level. Due to this substantial distinction compared to other decision systems that are based on predictive modeling of precisely observable outcomes, the case of insurance premiums must be studied separately.

To provide practitioners with principled indications for choosing a fairness objective, we investigate the fairness of insurance premiums on moral grounds and in terms of possible technical implementations. In this work, we apply group fairness criteria to the particular context of insurance premiums and evaluate them normatively. To do this, we draw on insights from the algorithmic fairness literature and explicitly consider moral arguments that can be given for or against them in this context. This allows us to argue for a specific conception of what is morally fair in the insurance context. As we will see, there is an already existing definition of fairness – namely, *sufficiency* – which is appropriate to measure the fairness of insurance premiums. We elaborate on the technical implementation of this definition to enable insurers to effectively mitigate the unfairness of insurance premiums. In addition, we conceptualize different degrees of personalization for risk models used to determine insurance premiums and normative arguments relevant to personalization levels.

## Developing Fair Personalized Insurance Premiums

In this section, we show how group fairness criteria can help build personalized risk models that are not unfairly discriminatory.

### Group Fairness

Let us start by introducing the concept of *group fairness* in general. Group fairness – sometimes also referred to as non-discrimination or statistical fairness – requires that benefits (or harms) that arise from the outcome of an algorithmic decision are distributed fairly across specified groups (Barocas and Selbst, 2016). These groups are specified with a so-called sensitive attribute (also called protected attribute). What constitutes such a fair distribution must be defined with an appropriate group fairness criterion. Several different mathematical measures have been proposed (Narayanan, 2018; Verma & Rubin, 2018).

According to Barocas et al. (2019), group fairness criteria typically fall into one of three categories: *independence*, *separation*, or *sufficiency*. *Independence* compares decision rates, whereas *separation* and *sufficiency* compare different types of error rates across groups. These notions of fairness are mathematically incompatible (Kleinberg et al., 2016; Berk et al., 2021). Therefore, one of the criteria must be chosen over the others.

### Actuarial Fairness and Solidarity

In the following, we will see that a well-known definition of fairness in the insurance context (*actuarial fairness*) is a theoretical construct that is not practically applicable for assessing the fairness of personalized risk models. We introduce some notation to look at the insurance case in more detail – for the sake of exposition, we assume a model for annual premiums:

- $\vec {x}$: describes the feature space for individuals, here, policyholders. It is used as an input for risk models.
- *A*: denotes the sensitive attribute which may or may not be contained in $\vec x$. For simplicity, we consider two groups: $A = \{0,1\}$. However, our findings can be applied to cases with more groups without substantive loss of generality.
- $Y_i$: denotes individual *i*’s annual sum of claims (when there is no need to refer to the individual explicitly, the subscript is dropped and only *Y* is written). Such a specific event can be seen as a realization of a random variable, i.e., a measurable outcome.
- $\mathbb {E}(Y_i)$: denotes an individual’s risk, which is the expected value of the individual’s annual sum of claims. This is the actual target variable that the ML algorithm estimates. $\mathbb {E}(Y_i)$ is well-defined for every individual. However, it cannot be measured on an individual level in reality.
- *D*: is the decision that is taken based on the algorithm’s estimation. It is the insurance premium that has to be paid by an individual.

In this context, when we say insurance, what we mean is, strictly speaking, private insurance between market actors that is not supported by moral principles^13^ and rests on considerations of mutual advantage alone. Private insurance, in this sense, always supports or generates the value of *solidarity* understood as *chance* solidarity: individuals within a group facing similar risks agree to share the costs resulting from a chancy event striking against the least fortunate of them. Chance solidarity is the logical consequence of risk pooling, where individuals with the same risks share future damage costs. Notice that, according to Lehtonen & Liukko (2011), there are two other types of solidarity in insurance: income solidarity and risk solidarity, which are both a form of subsidizing solidarity (as initially defined by Thiery & Van Schoubroeck (2006)): the former implements a subsidy that favors those with “meagre means,” the latter implements a subsidy that favors high-risk individuals (Lehtonen and Liukko 2011, p. 39). As we shall see briefly, solidarity as a *value* is not *needed* as a distinct moral motivation for a mechanism of chance solidarity to emerge if individuals are averse to risk to a sufficient degree and some agent (i.e., the insurer) is able to provide the mechanism that enables the sharing of the costs. In fact, private insurance usually implements pure chance solidarity (Lehtonen & Liukko, 2011) (which is part of any form of insurance), which is why we omit other types of solidarity for our moral argument.

What is, then, the point of (private) insurance? Insurance is the free exchange of risk between two agents: the insured and the insurer. The insured faces a risky prospect of an event of which, at most, the probabilities are known to him, and often not even those. Because the future occurrence of the event is unknown, the actual (dis)value of the risk can only be expressed as an expected (dis)utility. Such disutility may not be known as a precise or even properly approximate quantity to the insured, but this is obviously not a necessary hindrance to the exchange as long as one of the two parties (typically, the insurer) is able to produce such an estimate and it is trusted enough to do so. Usually, the two parties are asymmetrically situated with respect to the uncertainty of the risk insured against making the trade beneficial for both parties. While the insured faces uncertainty regarding the occurrence of an event that would cause some disutility, the insurer is able to measure the faced risk (Knight, 1921). Compared to individuals whose insurance claims are unknown in the beginning, the insurer’s loss is more predicable (in relative terms) as it represents an aggregation of a large number of small independent losses (Ohlsson & Johansson, 2010; Wuthrich, 2020).^14^

From the insurer’s point of view, who manages a broad portfolio of similar risk events, signing a large number of contracts, the final result over a large number of similar cases is indeed predictable within a narrow margin.^15^ If insurance is the trading of risk between agents who are significantly differently situated with respect to their ability to manage the risk, an intuitive standard of fairness is that what each party obtains in the exchange is of similar value. This leads to the idea of *actuarial fairness* – a notion that first appeared in Arrow (1963, p. 960) and has been present in academic literature ever since –, where the two parties, the insurer and the insured, exchange something (a premium for the insured, the expected loss for the insurance) which can be described as being of equal value. Using the mathematical concept of expectation values, the exchange between insured and insurance can be described as a kind of equivalence. Formally, we can write:

1 $$\begin{aligned} \mathbb {E}(Y_i) = D_i, \text { for all } i \in S, \end{aligned}$$

where *S* denotes the set of all individuals who buy insurance. For any individual *i*, the paid premium $D_i$ must equal this individual’s risk $\mathbb {E}(Y_i)$.^16^ An individual *i*’s risk is defined as the expected value of the future claims that this individual causes. This condition is equivalent to the idea of *actuarial fairness* expressed as *“individuals should pay premiums that reflect the risks they bring to the insurance pool”* (Landes 2015, p. 520).^17^ Thus, an insured person’s risk ($\mathbb {E}(Y_i)$) has traditionally been assumed to be what justifies the price of a premium an individual has to pay.

*Actuarial fairness* is defined at the individual level (Arrow, 1963; Landes, 2015).^18^ Dolman & Semenovich (2018) extend this to the group level by introducing a new fairness criterion they call *group actuarial fairness*. This criterion requires that the premiums are expected to be the same for individuals with the same risk, regardless of their group membership, and that they equal the average of the expected losses for the different groups, weighted by the sizes of the groups. Formally, *group actuarial fairness* is satisfied for two groups $A=\{0,1\}$ if:

2 $$\begin{aligned}{} & {} \mathbb {E}(D|\mathbb {E}(Y),A=0) = \mathbb {E}(D|\mathbb {E}(Y),A=1)\nonumber \\= & {} \frac{\mathbb {E}(Y_i|A=0) |\{i,A = 0\}| + \mathbb {E}(Y_i|A=1) |\{i,A = 1\}|}{|\{i,A = 0\}| + |\{i,A = 1\}|}, \end{aligned}$$

where $Y_i$ denotes the stochastic loss of individual *i*, and $|\{i,A = a\}|$ is the cardinality of the set consisting of individuals whose attribute *A* equals the value *a*. However, in practice, in the case of insurance pricing, it is unlikely that $Y_i$ corresponds to $\mathbb {E}(Y_i)$ exactly. Furthermore, an individual’s risk $\mathbb {E}(Y_i)$ cannot be measured because, at the individual level, we can only measure the outcome, which is influenced by chance. Let us clarify this by explaining the problem of the reference class.

Knowing the statistics of some events that actually happened in the past, the likelihood of an event can be predicted, but only over a population. While it is possible to predict an event – for example, if an accident will happen or not – when looking at a population, it is not possible to know exactly for which of the population’s individuals it will happen. Hence, an individual’s risk can then be defined by associating a probability with these statistics based on the aggregated data of a large pool of individuals. However, the data on which an insurer relies to compute risk are by necessity limited by the fact that the insured only has limited knowledge of its clients. But, moving away from *perfect individual premiums* (as would be the case for *actuarially fair* premiums), it is possible to compute the risk of an individual *qua* representative of a broader class. This implies that an insurer will never be able to compute the risk of an individual qua specific individual, but always as an individual characterized in a certain way. This is, of course, merely a statement of a classical problem in probability, namely the problem of reference classes. In its classical formulation by Reichenbach (1971, p. 374), this is the problem that: “If we are asked to find the probability holding for an individual future event, we must first incorporate the case in a suitable reference class. An individual thing or event may be incorporated in many reference classes, from which different probabilities will result.” For example, an insurer who only asks for the client’s age (in years) will compute the risk of the individual treated as a representative of the reference class of people born in the same year. This differs from the risk of the individual treated as a representative of the reference class of men.

Conditioning on reference classes instead of individual risk would represent a weaker form of *group actuarial fairness*. Since *group actuarial fairness* appeals to individual risk, it is also not possible to test whether *(group) actuarial fairness* is met or not. So, instead, one would need to rely on an estimate of the true individual risk $\mathbb {E}(Y_i)$. This poses the question of what reference class is morally appropriate to condition on for the evaluation of *group actuarial fairness*. From an insurer’s perspective, an approximation of *group actuarial fairness* as defined in Eq. 2 would be to group individuals that appear to have the same risks, i.e., forming groups based on the available data. For example, suppose an insurer defines premiums based on two features age and gender. Thus, from the insurer’s perspective, reference classes could be built using age and gender. However, these are not reference classes that should be considered from a fairness perspective because those are the reference classes that are in the insurer’s understanding of setting prices anyway. Any two individuals having the same age and gender pay the same premium. Thus, *group actuarial fairness*, understood as the expected premiums across groups *A* conditioning on any values for age and gender, is trivially satisfied. That is because any two individuals equal in the feature space are given the same premium, as the insurer cannot differentiate them based on the available data, making a comparison of premiums across groups *A* superfluous. An insurer could easily use some features to calculate risks and others to define reference classes for the fairness analysis (or just group some of the features used for the risk calculation to determine reference classes). For example, the insurer could calculate premiums simply based on a client’s age and build reference classes based on the gender of clients. Within those reference classes, there might be a difference between the average premiums across groups (i.e., conditioning on the reference classes instead of individual risk $\mathbb {E}(Y_i)$): groups of different genders only pay equal expected premiums if the age distributions of both groups are equivalent, as premiums are generated based on age alone in this example. However, this would be ad-hoc and not capture fairness in an intuitive sense. Instead, what would capture fairness intuitively is the consideration of true individual risks (representing individuals’ actual contributions to the risk pool – which cannot be measured), as specified in Eq. 2. Any alternative to that must involve an exogenous moral input relative to the needs of insurers, societal concerns, as well as the data available.

Thus, *actuarial fairness* and *group actuarial fairness* do not serve as a mathematical criterion to assess the fairness of personalized risk models, but they are rather to be used as the underlying moral perspective of certain insurance practices (i.e., the view that policyholders should pay premiums reflecting the risk they bring to the pool – instead of, for example, the alternative viewpoint that everyone should pay equal premiums).^19^

For these reasons, we need another definition of fairness that is both morally appropriate and practically applicable to the insurance context. For this purpose, we draw on the literature on fair ML and elaborate on the link between proposed fairness criteria and the insurance case.

### Application of Group Fairness Criteria to Insurance Premiums

We now investigate the potential of methods provided by the fair ML literature to measure systematic discrimination of certain groups for the case of personalized prediction models in the insurance context. In particular, we apply the widespread ML fairness criteria *independence*, *separation*, and *sufficiency* to the context of insurance premiums. We explain the connection to *actuarial fairness* for all three criteria and ask, for each of them, what normative argument can be offered in support and whether they are testable in ordinary conditions.

#### Independence

Consider, first, the idea of a group fairness criterion called *independence* – also known as *statistical parity* or *demographic parity* – in the ML literature (Barocas et al., 2019). Unlike *separation* and *sufficiency*, which compare error rates across groups, *independence* focuses on decision rates across groups. For a simple case of just two groups, *independence* can be formalized as:

3 $$\begin{aligned} \mathbb {E}(D,A=0) = \mathbb {E}(D,A=1). \end{aligned}$$

This means that individuals should pay the same premiums on average across groups. Compared to other notions of fairness, *independence* does not build on the choice of any risk-related reference class and only compares the premiums paid across groups *A*.

Recall the assumption we stated in the introduction that insurance, as we conceive of it, does not require risk solidarity, as opposed to mere chance solidarity. From this it immediately follows that *independence* is not an appropriate criterion to assess the fairness of personalized risk models in the context of insurance premiums if groups have different average risks. For such groups, *independence* (requiring equal average premiums across groups) would implement some form of risk solidarity,^20^ thus contradicting our assumption. Moreover, note that *independence* is incompatible *in most cases* with the idea of Aristotelian fairness, which, according to the classical formal definition of justice found in Ancient Greek philosophers, requires to treat like cases alike (Aristotle 1984a, V.3. 1131a10-b15; 1984b, III.9.1280 a8-15, III. 12. 1282b18-23), assuming “like cases” to denote “cases of like risk” in this context. *Independence* can be seen as a relaxation of *community rating* – as it is usually called in the insurance sector – which describes the arrangement whereby an insurer offers equal premiums to every individual, irrespective of individual differences in risk levels. An example of this would be health insurance offered at the same price for every individual, irrespective of health status. That is to say: community rating logically entails *independence*, but *independence* does not imply community rating, that is, it can alsoe achieved in other ways. This is also true for Aristotelian fairness, that is to say, community rating implies Aristotelian fairness, but Aristotelian fairness does not necessarily require community rating.^21^ Thus, *independence* and Aristotelian fairness are not strictly speaking incompatible. One illustration of this is that they are both satisfied by community rating, which guarantees the same premiums for all individuals and all groups. But, outside community rating and outside those cases in which all groups under consideration have exactly the same risks on average, *independence* can only be satisfied by violating Aristotelian fairness, and it requires risk solidarity at the level of both individuals (i.e., low-risk individuals subsidize high-risk ones) and groups (i.e., low-risk groups subsidize high-risk ones).

We will now provide an argument against *independence* in the context of insurance premiums on the basis of Aristotelian fairness. In particular, we will show that we can achieve both by applying community rating. We will then argue that we must either give up on Aristotelian fairness or *independence* since community rating cannot be required for private insurance. Since Aristotelian fairness is a more intuitive and established view of fairness than *independence*, we will conclude that *independence* must be rejected as a criterion of fairness.

Now, let us explain why we can only achieve Aristotelian fairness and *independence* with community rating and how we can solve the moral dilemma this entails. Suppose we assume that the relevant likeness in the insurance arrangement is the expected cost of being insured, as seems plausible for an exchange of goods between privates. In that case, the insurer who asks a higher price to a higher-risk individual does not violate Aristotle’s classical definition of fairness. Moreover, to satisfy *independence* without violating the classical definition of fairness, the insurer would be forced to adopt community rating, at least in some instances. For example, if we have two low-risk and one high-risk individual in group A and two high-risk and one low-risk individual in group B, the only way to satisfy *independence* (without treating like cases differently) is to treat low- and high-risk individuals in the same way.^22^ Hence, if average risk differs across groups and *independence* must be satisfied across groups, the only way to treat like cases alike would be to treat all cases alike, irrespective of their risk. However, the idea of community rating is unsuitable for insurance as a good provided privately on the basis of mutual advantage and in the absence of coercion by the state.

We briefly address the objection that if *independence* can always be achieved by asking the same premiums from all clients (which amounts to requiring community rating), this is what a fair insurer is supposed to do in every case. There are plausible arguments that indicate that an individual insurer in a competitive market cannot commit to community rating. The problem such an insurer would encounter is that (rational) low-risk individuals cannot be assumed to be willing to purchase insurance at the same price point at which high-risk individuals will. Hence, this insurer faces the concrete risk of ending up with a pool that only contains the individuals with the highest risk, which may be impossible to insure. Hence, community rating requires the state to legislate the insurance for the low-risk people at community rating prices. That is, it also requires coercing the low-risk people into purchasing insurance while paying a premium they would not be willing to pay for insurance without coercion. This coercion can be morally justified in some instances, but in other cases, it is simply not morally plausible and politically feasible. For example, many states provide health insurance to everyone, funded by general taxation, because, it may be argued, there is an obligation of justice that society covers citizens’ health needs (Daniels, 1981). This is an arrangement in which the state subsidizes the health insurance costs for the high-risk groups by requesting the low-risk groups to pay in taxes more than they would be expected to pay based purely on their risk, so this can cover the higher expected expenses of the high-risk individuals. Alternatively, the state requires that everyone purchases basic insurance that insurers are only permitted to sell at a uniform price.

We now face the dilemma of choosing between *independence* and Aristotelian fairness since we exclude the possibility of community rating for private insurance. First, we believe that the latter is a more intuitive and established view of fairness, which provides an argument against *independence*. Second, requiring that the expected premium paid by different groups be, on average, the same is also arguably morally inadequate since it ignores the possibility that this is based on the fact that one group has an average risk that is higher than the other group. In comparing average premiums across groups, without adding any further qualification (e.g., that the groups are made of individuals of similar risk), we are, therefore, not comparing similar with similar, and fairness does not require that dissimilar cases are treated similarly. Therefore, we conclude that *independence* is not an appropriate criterion to assess the fairness of personalized risk models.^23^

Notice that – in addition to the incompatibility with Aristotelian fairness – *independence* is also not compatible with *actuarial fairness* for groups with different average risks. This can be proved with a simple example. Suppose there are two groups, A and B, and that individuals in group A have a lower risk than those in group B, on average. To satisfy *actuarial fairness*, every individual must pay a premium equal to his or her risk. This implies that group A individuals pay lower premiums than those in group B, on average, and violates *independence*. Hence, *actuarial fairness* and *independence* are two contradicting notions of fairness unless insured individuals of different groups have exactly the same risk, on average. *Actuarial fairness* is a stronger constraint than Aristotelian fairness in that the latter, but not the former, is always satisfied by community rating. For *actuarial fairness* requires not only that *like* cases be treated alike but also that *different* cases be treated differently.^24^

#### Separation

Consider now the fairness standard of *separation* (also called equalized odds (Hardt et al., 2016), conditional procedure accuracy equality (Berk et al., 2021), or avoiding disparate mistreatment (Zafar et al., 2017)), formally defined as:

4 $$\begin{aligned} \mathbb {E}(D|Y=y,A=0) = \mathbb {E}(D|Y=y,A=1). \end{aligned}$$

This requires that among individuals with the same true outcomes (e.g., adverse events), the average premium be the same across the different socio-demographic groups. Notice that Eq. 4 conditions on the individuals’ realized damages *Y* and not on the individuals’ risks $\mathbb {E}(Y)$. Again we appeal to the point of insurance as a social practice – the reasons that make it useful and desired – to show that this standard is also inadequate, both morally and economically. The very point of insurance is the pooling of risks: all forms of insurance necessarily achieve some form of chance solidarity, at a minimum. So, the harm should be spread across the participants to the arrangement in a way that is independent of what the individual outcomes insured against turn out to be. If anything reasonably justifies a difference in premium, this is not whether the actual outcome insured against occurs.^25^

Given this premise, it is morally absurd to select, as a measure of the fairness of insurance, the criterion that requires similar average expected premiums for groups that distinguish between the actual positives and the actual negatives – e.g., those high-risk drivers that turn out to in fact have accidents vs. those high-risk drivers that turn out not to have any. In other words, *separation* describes as *fair* an algorithm assigning radically *different* expectations to individuals who can retrospectively be shown to belong to different classes in terms of their actual accidents.

However, the moral foundation of insurance is entirely antithetical to the idea that individuals should be treated differently based on the outcomes that actualize (except, as already discussed, when the already actualized outcomes have implications for the risks of future outcomes).^26^ The choice to insure drivers implies the rejection of the principle that costs ought to be allocated entirely based on responsibility for outcomes. If the principle of responsibility had been followed, insurance would not have been used, and chance solidarity would have been denied. Moreover, it would be absurd to require the large class of drivers who, say, do not have any accidents in nine years of driving to pay zero for their premiums.^27^ The only responsibility principle that is plausible in the insurance context is the principle of responsibility for risk (as opposed to outcomes).^28^ However, responsibility for risk may depart from actual risk, e.g., some individuals are bearers of risk for which they cannot be regarded as morally responsible (Dworkin, 1981; O’Neill, 2006; Daniels, 2004; Dolman & Semenovich, 2018; Dolman et al., 2020). Thus, the principle of responsibility for risk requires, in some instances, risk solidarity, i.e., low-risk individuals should morally subsidize high-risk individuals who are not fully responsible for their higher risk levels. However, as anticipated, we focus here on private insurance schemes in which risk solidarity is not socially expected, as a rule, or feasible.

*Separation* requires that for each (socially salient) group, the expected premium conditional on a given amount of claims should be the same. In contrast, *actuarial fairness* requires individuals to pay premiums representing their risk. As reported claims are just events that are not equivalent to risk, we most certainly end up with individuals who are equal in reported claims but whose risk differs – maybe even substantially. More specifically, if the true underlying distribution of risk is unequal for the considered groups, a perfect individual risk predictor is expected to violate *separation* – representing a phenomenon also known as the problem of infra-marginality (Ayres, 2002; Simoiu et al., 2017; Corbett-Davies & Goel, 2018; Hedden, 2021). Hence, the group fairness criterion *separation* does not comply with *actuarial fairness*.

An alternative definition of *separation* that conditions on $\mathbb {E}(Y)$ would make more sense from a moral point of view. Dolman & Semenovich (2018) call this alternative version of *separation* *group actuarial fairness* because it is defined as equal premiums for individuals with the same risk $\mathbb {E}(Y)$, on average across groups denoted by *A* (see Eq. 2). However, as we explained above, conditioning on $\mathbb {E}(Y)$ is difficult (if not impossible) because an individual’s risk can never be observed in practice.

Another option would be to come up with some similarity score to be able to group individuals with the exact same risk instead of actually measuring the risk for each individual. The most straightforward approach would be to group individuals with the exact same feature vector $\vec x$. However, this is useless in practice for two reasons: First, a prediction algorithm will automatically yield the same premium for such individuals. Therefore, this does not lead to a practical test of fairness. Second, with the vast amounts of data that insurers have for their clients, it is very unlikely that two individuals actually have the exact same $\vec x$. Even an approximation of such a score – in which individuals with risks that lie within some range would be grouped – is difficult to define.^29^

#### Sufficiency

A third definition of group fairness that is often discussed in the ML literature is *sufficiency* – also called *predictive parity* (Chouldechova, 2017) or *positive predictive value (PPV) parity* (Baumann et al., 2022) in the case of binary decision making systems.^30^ Hereinafter, we will argue for *sufficiency* as the most morally appropriate standard for private insurance without risk or income solidarity. To begin with, let us first analyze the relationship between *sufficiency* and the traditional view of fairness in the context of insurance premiums, which is *actuarial fairness*.

Formally, the group fairness criterion *sufficiency* can be expressed as:

5 $$\begin{aligned} \mathbb {E}(Y|D=d,A=0) = \mathbb {E}(Y|D=d,A=1). \end{aligned}$$

*Sufficiency*, as defined here, is equivalent to *well-calibration* (Chouldechova, 2017) and very similar to the criterion of *calibration within groups*, which is one of the criteria considered by Kleinberg et al. (2016); Hedden (2021) (as they both condition on *D*). The only difference compared to *well-calibration* is that here we consider the paid premium instead of the received risk score. *Calibration within groups* is a stronger variant of *sufficiency*: for the insurance case, this would require that for each possible premium *d*, the expected damages of individuals paying that premium must be the same for each relevant group, and it must be equal to the paid premium. Formally, we can write: $\mathbb {E}(Y|D=d,A=0) = \mathbb {E}(Y|D=d,A=1)=d$.^31^ Notice that while the fairness criteria *independence* and *separation* fall prey to the problem infra-marginality, the *sufficiency* criterion does not (Ayres, 2002; Simoiu et al., 2017; Corbett-Davies & Goel, 2018; Hedden, 2021). Further, notice that compared to *group actuarial fairness*, the fairness criterion *sufficiency* conditions on a reference class that is measurable, i.e., the paid premium.

For sufficiently large group sample sizes, the expected loss $\mathbb {E}(\mathbb {E}(Y_i)|D=d,A=a)$ for individuals of a group *A* who paid a premium *D* approximates the mean of the actually observed damages $\sum \frac{Y_i}{N_{D=d,A=a}}$ of those individuals:

6 $$\begin{aligned} \mathbb {E}(\mathbb {E}(Y_i)|D=d,A=a) \approx \sum \frac{Y_i}{|\{i,D=d,A = a\}|} \end{aligned}$$

$|\{i,D=d,A = a\}|$ is the cardinality of the set consisting of individuals that received the decision *d* and whose attribute *A* equals the value *a*. Under the assumption that premiums were set *actuarially fair* ($\mathbb {E}(Y)=D$), individuals who have the same risk should pay the same premium and vice versa. Therefore, we do not expect the average observed damage to differ across groups when conditioning on *D*. Hence, *actuarial fairness* implies *sufficiency*. However, the inverse and the converse statements are not logically true: If premiums are not *actuarially fair*, *sufficiency* might still be satisfied. Similarly, if *sufficiency* is satisfied, premiums are not inevitably *actuarially fair*. In particular, there might be a systematic bias against some individuals, but if their claim costs *Y* even out due to aggregation, *sufficiency* might still be satisfied.

In real data samples, the equality is not strictly met due to the statistical variability of the observed damages, which we have to consider. For two groups $A=\{0,1\}$, a natural way to test for equality is a statistical test,^32^ based on the following Null hypothesis:

- $${\textbf {H}}_O$$: $$\mathbb {E}(\mathbb {E}(Y)|D=d,A=0) = \mathbb {E}(\mathbb {E}(Y)|D=d,A=1)$$
- $${\textbf {H}}_A$$: $$\mathbb {E}(\mathbb {E}(Y)|D=d,A=0) \ne \mathbb {E}(\mathbb {E}(Y)|D=d,A=1)$$

We can test the null hypothesis ${\textbf {H}}_O$ by performing a statistical test based on the observed damages. However, failing to reject the null hypothesis does not imply that the null hypothesis is accepted. For, a lack of evidence to conclude that the effect exists does not prove that the effect does not exist. Hence, it is impossible to statistically prove that there is no difference in risk between individuals from two groups who pay the same premium. Instead, failing to reject the null hypothesis in a statistical test usually simply indicates that the data does not provide sufficient evidence to conclude that there is indeed a difference between groups. A reason for this might be that the sample size is too small or that the variability in the data is too high, so the effect cannot be detected.

Note that testing this hypothesis is equivalent to testing whether *sufficiency* is satisfied. Based on this test, if we reject the null hypothesis in favor of the alternative hypothesis ${\textbf {H}}_A$, we can conclude that prices are not *actuarially fair*. In other words, group membership affects the observed outcome after controlling for the paid premium. So, if *sufficiency* *is not* satisfied with regard to the premiums paid across groups, then we know that prices are not *actuarially fair*. But, if *sufficiency* *is* satisfied across groups, it does not necessarily mean that prices are *actuarially fair*. It is very well possible that prices are not *actuarially fair* even though *sufficiency* is satisfied. For example, if the risk of individuals paying a specific premium is the same across groups on average but not on an individual level. In this case, *sufficiency* is satisfied even though premiums are not *actuarially fair*.

We argue that *sufficiency* is an appropriate measure of fairness across groups in the context of insurance premiums, even if it does not entail *actuarial fairness*, as shown above. This extends the above discussion of *sufficiency* in that it is not merely useful to test for violations of *actuarial fairness*. The premise for which we argue is rather minimal. We propose that we conceptualize the fairness of insurance by asking what constitutes an unfair (in the sense of discriminatory) treatment *among individuals who pay the same premium*. So, we argue that the correct viewpoint is to start with the actual difference that is both observable and in need of a moral justification, which is the difference in premiums between individuals. If we start from this observation, it is natural to ask whether the people who pay the same premium obtain the same advantages by virtue of doing so, or whether the advantage they obtain is related to their group, for example, gender. In order to provide a justification of *sufficiency* that is independent of *actuarial fairness*, we shall simply assume the following: if clients who commit the same resources to insurance obtain unequal advantages in a way that is less favorable to group A relative to another group, B, that is an instance of (indirect) discrimination against A and in favor of B on account to the group that A belongs to, but B does not belong to.^33^ Therefore, we shall ask: what is the advantage people receive from insurance, and how should this be measured? The first answer is that the advantage for an insured individual is that, when the adverse outcome occurs (for example, one is liable for the damages of a car collision), the insured will not pay the damage out of pocket, but instead, this will be covered by the insurer. In other words, counter-intuitively, the benefit of insurance is the mathematical expectation that the insurance will pay that certain sum that the insured individual would have been required to pay out of pocket if he or she had not been insured. Thus, the benefit can be measured as the mathematical expectation that the harmful outcome insured against happens. For example, for the individual who buys coverage against liability for a car collision, the expected benefit equals the risk of a car collision, namely, the amount of damage the insured would otherwise be required to pay times the probability of this happening.

Now that we know what the advantage is and how to measure it, we can specify more rigorously what it means, across individuals who pay the same premium, to receive favorable or unfavorable treatment in exchange for that premium on account of membership to a specific socio-demographic group. Suppose that, among people who pay, for example, $800 in annual premium, women have, on average, expected claims of X, and men expected claims of 2X. Intuitively, men are unfairly benefited because, as men, they have higher expected benefits for purchasing insurance at the same price women do. This could be comparable to a baker selling twice the amount of bread to men than to women in exchange for the same amount of money; in other words, it is comparable to a baker selling bread at higher prices to women than to men, which is certainly discriminatory if anything is.^34^ Therefore, it is fair that people who pay the same premium have the same expected benefit in terms of what they pay it for (that is, risk coverage) on average in a way that is statistically independent of the group they belong to (e.g., whether they are men or women). Thus, requiring no unfair advantage related to membership to specific groups, in the insurance case, is equivalent to requiring that, *on average*, people who pay the same premium should have the same risk (insured against), meaning, the same expected loss, independently of the group (e.g., men or women) they belong to.

This view is similar (and, as we explained above, mathematically related to) *actuarial fairness*, but it is not identical. *Actuarial fairness* is the view that the risk (the expected harm from the collision) should be equal (as an expected value) to the premium paid. The intuition here is to compare the expected benefit of the insured with the price actually paid to the insurance and require that they are equal in expectation in a fair exchange. Any further criterion of equality – say between groups – may follow logically, but it is morally derivative from a view of the fairness of this insurer-client relation. *Sufficiency* – the criterion we invoke – is different. *Sufficiency* does not require the client’s expected benefit (again, the risk insured against) to be equal to any specific value.^35^ A fortiori, it does not require it to be equal to the premium paid for the insurance, which is what *actuarial fairness* asks. *Sufficiency* only requires that the expected benefit (that is, the risk) for clients paying the same premium, whatever that is, should not vary in a way that statistically depends on the morally salient groups.

Notice that we consider the risk of the individual to be a mathematical expectation, calculated as an average value for the outcome (e.g., the amount of damage) across a population. We shall briefly explain why it is this average value that matters morally, and justifiably so. We shall do this as a reply to those that would object that it should be the individual risk, not some kind of average risk, that determines what the expected benefit is for each person who is insured. Such an objection would concede that fairness requires the expected risk to be statistically independent of group membership, conditional on paying a given premium, but complain that the risk should be computed on a strictly individual level, which is not what *sufficiency* entails. Our reply is that an insurer (e.g., an insurance company) lacks the ability to determine risk at the individual level.^36^

Too fine-grained groupings are not desirable for a fairness assessment^36^, which means that more personalization than the usual coarse demographic groups (e.g., comparing men and women, white and blacks, or the resulting intersectional groups) is often not possible in terms of testing for *sufficiency*.^37^ Hence, to make meaningful comparisons for fairness, we need to stick to groups of a certain size, allowing probability to be measured in practice.

We conclude that the group fairness criterion *sufficiency* is not only a test for the violation of *actuarial fairness* but actually a morally appropriate measure of fairness in the insurance context. It allows us to exclude with statistical significance that some group is systematically disadvantaged. Therefore, testing whether models satisfy *sufficiency* helps detect systematic unfairness of insurance premiums across groups.^38^

Furthermore, instead of testing for *sufficiency* for any morally arbitrary groups, the values of *A* must be an exogenous input that is determined by some theory reflecting the social and moral concerns of society as well as the needs of insurers. However, providing detailed moral heuristics to determine the groups to consider lies outside the scope of this paper.

**Fig. 1:**
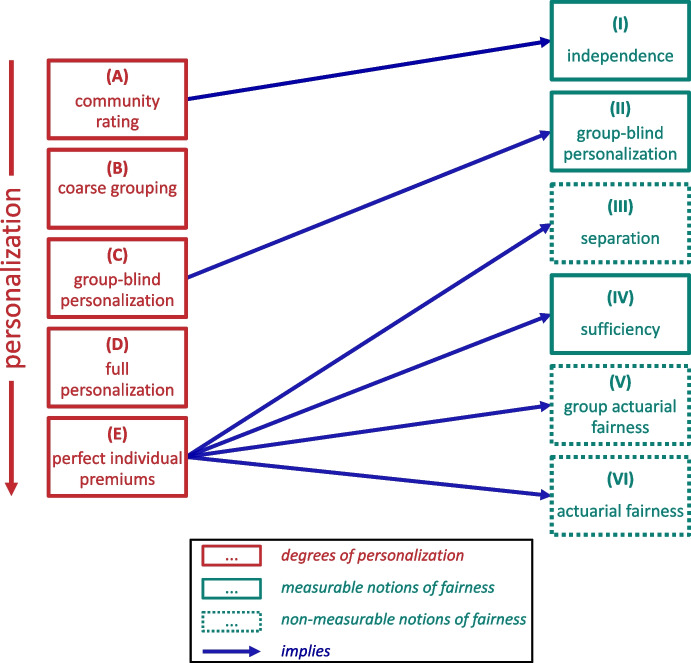
Degrees of personalization and their implications w.r.t. different notions of fairness

### Fairness for Different Degrees of Personalization

Using a personalized risk model to set premiums requires the choice of how much personalization one wants to strive for (Cevolini and Esposito, 2020; Lindholm et al., 2022; Wüthrich and Merz, 2023). In various domains, companies nowadays target customers on a much more fine-grained level, aiming to collect more and more user data. This development is primarily attributed to the advances in ML technology in recent years. Similarly, insurance companies often use vast amounts of data to predict the risk applicants want to be insured against. As is also the case in other domains, insurers typically wish to extend the feature space $\vec x$ with as much data as possible in the hope of improving the accuracy of their risk models. In this endeavor, the degree of personalization is not predetermined but instead constitutes a choice that depicts the underlying moral values. The moral understanding of what represents a fair premium is somewhat related to the degree of personalization – as visualized in Fig. 1. In that regard, we now describe possible choices and, thereby, follow up on our argument for the imposition of the group fairness criterion *sufficiency* in the context of private insurance. Figure 1 visualizes five degrees of personalization:

(A) *Community rating*: No personalization at all can be achieved with community rating. If an insurance company does not use any personal information of the applicants and asks everyone the same price, learning a model to predict individual risk is superfluous.^39^
(B) *Coarse grouping*: Grouping individuals in a coarse-grained manner requires that an insurance company has some data on the applicants (as discussed more thoroughly in Section 3.3.3). However, this can be done following rules based on insurance mathematical considerations instead of learning a classifier. An example would be to require older people to pay a high price and young people to pay a low price to acquire life insurance – without personalizing the premiums on the basis of any other attributes.
(C) *Group-blind personalization*: Group-blind personalization (also called *fairness through blindness* or *fairness through unawareness*) is somewhat similar to our notion of coarse grouping. However, much more personalization is allowed here instead of requiring minimal personalization based on a set of specific features. The only restriction is for the prediction model to be blind with regard to a sensitive attribute *A*.
(D) *Full personalization*: As we explain in Section 3.2, insurers need to aggregate the information on historical losses over a population to estimate the risks of applicants accurately. Without any constraints, insurers can fully personalize the premiums by using all available data.
(F) *Perfect individual premiums*: From an actuary’s perspective, a perfect premium is one that is equal to an individual’s risk, which would require full personalization. Note that this would imply *actuarial fairness*. Knowing all information about every applicant – similar to a God-like perspective – would allow insurers to set perfect individual premiums without the need for aggregation. Perfectly predicting risk on an individual level is not feasible in practice.

Figure 1 additionally visualizes six different notions of fairness:

(I) *Independence*: *Independence* requires equal average premiums across groups, which amounts to some form of risk solidarity for groups with different average risks. Though, by virtue of the assumption that such a solidarity mechanism is not required in the case we focus on, we do not further elaborate on this and only mention it as a possible choice for cases that do not fall under this assumption. Note that while community rating (A) implies *independence* (as shown in Fig. 1), its converse is not true as there are many other pricing strategies that satisfy *independence* without setting the exact same premium for everyone. However, if (any larger degree of) personalization is preferred over community rating (A), *independence* is not an appropriate measure of fairness, as it would lead to a violation of Aristotelian fairness when groups have different average risks (see Section 3.3.1).
(II) *Group-blind personalization*: In practice, group-blind personalization is sometimes seen as a fairness criterion by itself to avoid disparate treatment (Dolman et al., 2020). Omitting the sensitive attribute for predictions is a very intuitive intervention that is also being promoted by the European Union.^40^ However, when we talk about fairness, it seems more meaningful to strive for a morally meaningful fair impact measure (Barocas and Selbst, 2016), as opposed to a mere procedural constraint. And for that aim, group blindness is not an effective measure. Requiring the sensitive attribute’s omission reduces personalization but does not ensure fairness, as it does not necessarily avoid indirect discrimination against disadvantaged groups. In fact, sensitive attributes can often be inferred from other information in the feature space (Barocas et al., 2019). Also, in the case of insurance, large datasets with highly correlated features are likely. Therefore, group-blind personalization is not an effective approach to ensure the fairness of risk models.
(III) *Separation*: The fairness notion *separation* is implied by perfect individual premiums. However, as described in Section 3.3.2, it is not measurable in practice.
(IV) *Sufficiency*: Imposing *sufficiency* as a fairness constraint is not compatible with community rating, assuming that groups do not consist of individuals with equal risks, on average. Hence, there is a choice to be made for setting premiums: Should individuals pay the same premiums or premiums that satisfy *sufficiency*? In this paper, we argue for the latter to be an appropriate measure of fairness of premiums across groups in the context of private insurance, where no income or risk solidarity is required, as outlined in-depth in Section 3.3.3. Hence, premiums are fair if individuals who pay the same premium produce equal claims in expectation across groups.^41^ Note that this concept is compatible with Aristotelian fairness but does not necessarily imply it. Further, note that perfect individual premiums (E) imply *sufficiency* (as shown in Fig. 1). Hence, at this end of the spectrum, *sufficiency* may be used as a test for *actuarial fairness*. The fairness notion of *sufficiency* is thus perfectly compatible with a perfect predictor of individual risk. At the other end of the spectrum, where individuals are simply grouped on a coarse-grained level (B) (potentially blind w.r.t. *A* (C)) or premiums are fully personalized (D), *sufficiency* can be seen as an additional constraint requiring that individuals who pay the same premiums, at least on average, end up with equal benefits across groups.
(V) *Group actuarial fairness*: In this paper, we provide an argument for *sufficiency* as a minimal fairness requirement for individual risk premiums in the insurance context. This is compatible with but not implied by *group actuarial fairness*. As explained in Section 3.2, *actuarial fairness* conditions on the true individual risk $\mathbb {E}(Y_i)$, which cannot be measured. However, we can still consider it in its weaker form, conditioning on multiple relevant reference classes – as potentially an approximation of the true individual risks. Requiring equality of expected claims across groups *A* for additional reference groups is possible as long as the resulting groups are large enough. Otherwise, there is no statistical significance for the inequality of average claims across groups. Doing this for all possible subgroups of *A* corresponds to a concept called *multicalibration* (Hebert-Johnson et al., 2018). For the case of insurance premiums, *multicalibration* is a promising approach to simultaneously satisfy *sufficiency* and *group actuarial fairness*, and it represents the closest one could get to *actuarially fair* premiums on an individual level. We refer the interested reader to Hebert-Johnson et al. (2018), who provide a theoretical approach to achieving *multicalibration*.
(VI) *Actuarial fairness*: The concept of *actuarial fairness* is equivalent to perfect individual premiums. Thus, it is incompatible with lower degrees of personalization. See Sections 3.2 and 3.3.3 for more details on perfect individual premiums and *actuarial fairness*.

Notice that *actuarial fairness* logically entails *group actuarial fairness*, *sufficiency*, and *separation*. However, *actuarial fairness* and *separation* are not measurable in practice, and *sufficiency* is preferable to *group actuarial fairness* for reasons outlined in Section 3.

Requiring premiums to satisfy *sufficiency* allows for different levels of personalization: (B) - (E). Following this notion of fairness, insurers can use available data to develop fully personalized prediction models as long as they are not biased towards a specific group, denoted by *A*. This allows insurers to set competitive prices by approximating actuarial rates while also ensuring non-discrimination of groups according to the sensitive attribute *A*. Even if premiums must be fair across groups, meaning that they must satisfy *sufficiency*, striving for a certain degree of personalization constitutes an additional choice. In practice, this is often predetermined by data availability because the more personalization is aimed for, the more data is needed. However, there exist various possible reasons for the lack of data, for example, because it cannot be produced. Also, preserving privacy may have a negative effect on personalization. Hence, strong privacy laws can also restrict the collection of specific data. However, even though data is available, one might still opt for community rating in certain situations. For example, there may be moral reasons in some specific cases (e.g., health insurance) supporting some degree of risk solidarity in addition to chance solidarity.

## Practical Example

Assume an insurance company’s goal is to offer an insurance product that covers third-party liability claims. The insurance thereby acts as an intermediary in the risk pooling process for risk-averse individuals. Even in pools of individuals of the same risk, individuals end up with very different claim costs due to mere chance, which is why the idea of chance solidarity is the rationale underlying the insurance activity (i.e., claim-free policyholders subsidize policyholders that request compensation for a covered loss). The insurance company bears for the overall riskiness of the pool (which decreases with an increasing pool size) as the sum of all claims paid by an insurance provider in a given year is not known in advance. To be able to provide their service, the insurance company has an incentive to estimate the likelihood of those uncertain future events. Therefore, the company’s goal is to predict the risk of prospective clients as accurately as possible in order to be able to offer personalized premiums that are in line with the risk that those individuals bring to the pool. Due to the uniqueness of all (prospective) policyholders, perfectly computing individual risk is not feasible. Instead, insurance companies rely on risk models trained with datasets consisting of current policyholders (whose caused claims are known as these individuals are already part of the insurance pool). Applying such a model to prospective policyholders (for which neither the risk nor the damages they caused in the past are known) allows for estimating those new individuals’ risks. Thereby, the model relies on an aggregation of claims of known policyholders as an approximation of risk.

As a showcase for our moral argument in favor of the group fairness criterion *sufficiency*, we analyze the fairness of insurance premiums based on the dataset *freMTPL2freq*, which is publicly available as part of the R package CASdatasets (Charpentier, 2014).^42^ The dataset contains risk features collected for 678,013 third-party liability policies (all in the same year).^43^ We applied a similar pre-processing as Lorentzen & Mayer (2020), mainly to remove outliers and to exclude duplicate instances.

We fit a generalized linear model (GLM),^44^ which is the standard method for individual pricing of non-life insurance products (Ohlsson & Johansson, 2010; Wuthrich, 2020). For simplicity, we assume that the insurance sets premiums based on the predicted risk (i.e., the pure premium, which is defined as the claim frequency times the claim severity (Ohlsson & Johansson, 2010)) and does not further adjust prices (e.g., based on market considerations). Furthermore, we assume a severity of 1 for all claims. Hence, we need to predict the claim frequency *Y* to estimate the risk. In this setting, the paid premium *D* represents the predicted risk $\mathbb {E}(Y)$. The Poisson GLM is fitted on 80% of the data (the other 20% are used for the fairness evaluation) to model the response variable *Y* *frequency*, which we define as $\frac{ClaimNb}{Exposure}$, as a function of the predictor variables (i.e., the feature space $\vec x$) VehPower, VehGas, DrivAge, logDensity, PolicyRegion.^45^ This model is unaware of the sensitive attribute. Thus, we call it a *blind* model.^46^

**Fig. 2:**
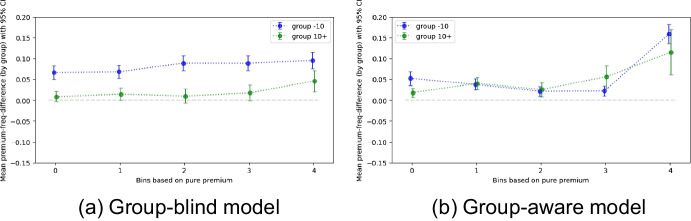
Measuring *sufficiency* for insurance premiums

Suppose that the age of the vehicle splits the population into two groups of individuals – those who own a car that is less than ten years old (group -10) and those who own a car that is at least ten years old (group 10+) – for which we want the model to be fair – as mentioned previously, we disregard the question of how to choose a specific attribute for which it is morally desirable to ensure group fairness. To test if the premiums are fair regarding these two groups, we must check for a violation of *sufficiency*.^47^ Figure 2a plots the fairness outcome based on the test set, consisting of the 20% of the data that have not been used to train the model. To measure *sufficiency*, we grouped the entire population into equally large bins based on the paid premium. The y-axis visualizes the average difference between claim costs and paid premiums per group for each bin. As can be seen, in this case, *sufficiency* is not satisfied. There is a statistically significant systematic disadvantage against individuals who have a car that is younger than ten years. Figure 2b visualizes the fairness for a second model for which the sensitive attribute is used as a predictive variable during training, thus, named the *aware* model. Compared to the *blind* model, the *aware* model is free of systematic differences between the two groups. Hence, adjusting the model has increased the fairness and resulted in a better performance (average unit Poisson deviance of 0.58 compared to 0.59 in the blind model).^48^

Here, we propose a simple method to change the model by including the sensitive attribute and show that this can lead to more fairness and better performance. However, these results depend on the applied model and the underlying data and are therefore not generalizable. In other instances, adding predictors can also lead to overfitting and may not increase fairness for cases with non-linear relationships. Therefore, it is important to mention that this is just one of the countless possibilities to adjust a risk model, so the above results must be taken with a grain of salt. This practical example shows that using a personalized risk model may be unfair for specific social groups. The question of how to provide a generalizable, optimal method to ensure the fairness of risk models remains an important open question. Berk et al. (2017) propose using fairness regularizers to ensure group fairness of regression problems. Other researchers have argued for a constrained minimization of the expected loss to uncover the accuracy-fairness frontier in regression problems (Agarwal et al., 2019). Steinberg et al. (2020a, 2020b) instead follow an information-theoretic approach, which relies on conditional probability density functions to approximate group fairness criteria in the regression setting.^49^

## Conclusion

It is widely acknowledged that biases are a common occurrence when prediction models are applied to humans across various application fields, including insurance. Therefore, special efforts must be made to avoid unfairness elicited by using personalized risk models used to determine insurance premiums. In this paper, we map group fairness criteria, which have emerged in the ML literature in the past years, to the context of private insurance. We argue that neither *independence* nor *separation* are appropriate measures of fairness, assuming that there is a difference between the average risk of groups that does not require compensation. Instead, we argue that the group fairness criterion *sufficiency* is morally appropriate for assessing the fairness of premiums in the context of private insurance involving only chance solidarity. By using *sufficiency* as a test to identify cases where an insurer systematically overestimates (or underestimates) the risk for some group (e.g., due to biases in the data used to generate the risk prediction model), it is possible to avoid systematic biases.

## Footnotes

1 *Independence* is also known as *statistical parity*, *demographic parity*, *equal acceptance rate*, or *group fairness* (Dwork et al., 2012; Zliobaite, 2015; Barocas et al., 2019). *Separation* is sometimes also referred to as *equalized odds* (Hardt et al., 2016), *conditional procedure accuracy equality* (Berk et al., 2021), or *avoiding disparate mistreatment* (Zafar et al., 2017) *Sufficiency* is also known as *well-calibration* (Chouldechova, 2017), *calibration within groups* (Kleinberg et al., 2016), *calibration by group* (Barocas et al., 2019), *conditional use accuracy equality* (Berk et al., 2021), or *positive predictive value (PPV) parity* in the binary case.

2 For a distinction of these types of solidarity, see Section 3.2.

3 Much of the existing academic literature on fair ML focuses on binary classification algorithms, such as criminal risk assessment (Angwin et al., 2016; Berk et al., 2021) hiring (Raghavan et al., 2020), college admission (Kleinberg et al., 2018), lending (Fuster et al., 2017), or social service interventions (Chouldechova et al., 2018; Potash et al., 2015). Few researchers have investigated the fairness of regression models with continuous target values. For example, Berk et al. (2017) and Steinberg et al. (2020a) both provide a solution that promises to ensure the fairness of regression models. While Berk et al. (2017) define fairness criteria in the form of regularizers, Steinberg et al. (2020a) follow an information-theoretic approach. Both provide technical solutions for specific definitions of fairness appropriate for regression models. However, they do not specify how to choose a definition of fairness that is morally appropriate for a specific regression model, which is a crucial preliminary step.

4 For example, Donahue & Barocas (2021) elaborate on the trade-off between the two competing views of fairness: *actuarial fairness* and solidarity. They show that it is possible to deviate from *actuarial fairness* in a way that strictly benefits everyone. This is the case because pooling reduces costs due to economies of scale. However, Donahue & Barocas (2021) follow a game-theoretical approach with just two groups: a high-risk group and a low-risk group. They do not consider group fairness with regard to a sensitive attribute (such as gender) at all.

5 Using many different variables allows for what we call a high *degree of personalization.*

6 Dolman et al. (2020) provide a high-level framework to answer this question of which rating factors should be used. Similarly, Loi & Christen (2021) discuss four *prima facie* non-consequentialist ethical objections against using four distinct types of factors.

7 See case C-236/09, *Association Belge des Consommateurs Test-Achats ASBL and Others v. Conseil des ministres* available at: https://curia.europa.eu/juris/liste.jsf?language=en &num=C-236/09.

8 See Section 3.1 for a description of sensitive attributes.

9 Hedden (2021, p. 214) refers to it as *calibration within groups* which is a very similar, though slightly stronger variant of *sufficiency*, defined as: For each possible risk score, the expected share of individuals belonging to the positive class should be the same across groups and equal to that risk score. See Section 3.3.3 for a formal definition of *sufficiency* and *calibration within groups*.

10 As Hedden (2021, p. 225) himself acknowledges “So far, I have given no positive argument in favor of Calibration Within Groups. It is just the only one left standing in the case of people, coins, and rooms.” Hedden goes on to add that: “it is intuitively compelling and easily motivated. If it were violated by some algorithm, that would mean that the same risk score would have different evidential import for the two groups.” (Hedden 2021, p. 225).

11 Notice that it would have *also* been coherent with Hedden’s paper to show that either one of *independence* or *separation* is the relevant criterion for fairness in a specific context. That is because one of them could have been necessary for fairness in this context, but not in all possible contexts.

12 Notice that our approach (as we will show in Section 3.3.3) corresponds to testing for a violation of *actuarial fairness* with some degree of approximation.

13 Other than the utilitarian principle interpreted as requiring profit maximization in competitive free markets, as argued by Friedman (1970, 2007).

14 When one writes “predictable” in this context, one does not mean exactly predictable, but rather predictable within a confidence margin, which can also be estimated.

15 Even if both parties have perfect knowledge of the objective chances of all possible events, it can be mutually beneficial for one party to buy insurance from another, e.g., due to the diminishing marginal utility of money (i.e., the utility curves of risk averse individuals have declining marginal utility) or due to the fact that individuals prefer to not lose what they already have instead of receiving something they do not yet have (i.e., individuals are loss averse) (Rabin & Thaler, 2001; Wakker, 2010).

16 This concept is also known as “pure premiums” in the actuarial literature.

17 Notice that *actuarial fairness* makes sense economically. If competition forces insurers to offer insurance at the lowest possible price, actuarial rates are the minimal premiums an insurance company can charge to avoid insolvency due to underwriting losses. Clearly, these rates are calculated by estimating the expected value of the future losses, which amounts to the *actuarially fair* price, as defined.

18 Notice that the most natural interpretation of the definition provided by Landes (2015, p. 520), saying that “individuals should pay premiums that reflect the risks they bring to the insurance pool,” is that every individual should pay a premium that reflects his/her risk brought to the pool. In fact, groups are not mentioned.

19 Of minor importance for our work is the fact that, in practice, the price individuals ultimately pay to buy insurance is not only based on their risk. Even if the company offering insurance intends to conform to the idea of *actuarial fairness* the premium *D* is usually some monotonic function $g(\hat{Y})$, where $\hat{Y}$ corresponds to an insurer’s estimation of the risk $\mathbb {E}(Y)$ (Dolman & Semenovich, 2018). In this regard, *D* also accounts for an insurer’s administrative costs and profits and not just for the cumulative risk of the participating individuals. Our findings generalize to this interpretation of *actuarial fairness*.

20 This can be proved with a simple example where we have two groups, A and B, and individuals in group A have a lower risk than those in group B, on average. To satisfy *independence* at least for one of the two groups, the average premiums do not correspond to the average risk, leading to risk solidarity (for example, if the low-risk group (A) pays premiums reflecting the high-risk group’s (B) average risks).

21 For notice that, if all individuals pay the same premium, *a fortiori* all individuals with the same risk also pay the same premiums. Thus, community rating satisfies *both* *independence* (all groups pay on average the same premiums) and Aristotelian fairness (all individuals with the same risks pay the premiums). But clearly, in principle, there are other ways to satisfy Aristotelian fairness other than community rating, violating *independence*. For example, *independence* is violated if every individual pays his or her *actuarially fair* risk and groups are composed of individuals of different average risks, which achieves Aristotelian fairness. This statistical phenomenon is known as the problem of infra-marginality (Ayres, 2002; Simoiu et al., 2017; Corbett-Davies & Goel, 2018; Hedden, 2021).

22 In theory, if there were an individual with a specific risk in one group and no individual with an equal risk in the other group, it would be possible to exploit this situation by using this individual as a wild card, who is offered a premium that balances out the difference in average premiums between groups to achieve *independence*. For example, assume that in the previous example, there is a fourth individual in group A with a very low risk and that group B remains the same (three individuals, none of which has a very low risk). The fact that there is no individual with a very low risk in group B allows us to set any premium for the very-low-risk individual in group A without violating Aristotelian fairness. Thus, the very-low-risk individual can be used as a wild card who receives such a low premium that, on average, premiums are equal for both groups. However, first of all, such behavior would be deeply unfair (either for the wild card, if this individual is overpriced, or for the rest, if the wild card is underpriced). Note that in the provided example, the very-low-risk individual would need to pay an exorbitant premium (which is even higher than the one of the high-risk individuals) for *independence* to be achieved. This idea has also been referred to as “gerrymandering” (Kearns et al., 2018) or subset targeting (Dwork et al., 2012). Second, in certain cases (for example, if there is only one individual that can be used as a wild card to balance out a very big difference in average group premiums – or a difference in average premiums between very large groups), it would be necessary to set negative premiums or infinitely high premiums to achieve *independence*. For these reasons, we neglect such possibilities for our argument despite its theoretical feasibility.

23 Notice that Loi & Christen (2021) also investigate the criterion *independence* in the context of insurance. However, they only investigate the emerging trade offs w.r.t. accuracy when enforcing *independence* and its consequences. They do not consider the possibility of enforcing sufficiency or *separation* as alternative operationalizations of fairness.

24 Our argument in this paper that insurance fairness does not require *independence* is coherent with FEC (“fair equality of chances,” which was introduced by Loi et al. (2019) and later extended by Baumann & Heitz (2022)), given the assumption that the feature J, that which justifies an inequality in expectations of utility, is the individual risk of the client, while the (dis)utility of the client corresponds to the premium paid.

25 In some cases, e.g., car insurance, it may look as if people pay a premium based on the actual outcomes; but this is an illusion, for given outcomes, e.g., a past accident, only affect the premiums that are paid as insurance for a *different outcome* i.e., the next possible accident. This is because the past outcomes affect the risk class of the individual, not because the individual is treated as responsible for that very outcome that has already occurred. So notice that, even in this apparent counterexample, the justification for the higher premium is *not* the individual moral responsibility for the actual accident, but rather the fact that this is indicative of a higher risk of future accidents.

26 Even in those cases in which social mechanisms designed to ascertain responsibility are in place, the existence of insurance implies a distribution that departs from one that would reflect individual responsibility *only*. Consider, again, the case of liability for car accidents. Here we have an institutional mechanism to ascribe responsibility for the damages in the case of an accident. But the costs of the accidents are not allocated in a way that fully reflects the responsibilities that have been identified. That would be the case if the driver were not insured, i.e., he or she would have to bear the full costs of the damage for which he or she is deemed liable alone. By contrast, if the driver is insured, other (ex ante similarly risky drivers) will share the costs, even though it is clear that those drivers share none of the responsibility for the accident (e.g., they do not share the moral blame, if any).

27 The sociologist Francois Ewald, in “The Birth of Solidarity” (Ewald & Johnson, 2020) has argued that the public acceptance of the insurance principle implied side-stepping the assessment of the accidents taking place during work in terms of responsibility. In the framework of individual responsibility, the interests of employers and their employees were in tension with each other, leading to continuous conflicts in relation to who was to be held accountable for the harm. In the insurance view, on the contrary, individual responsibility for the accident becomes irrelevant; the issue is side-stepped providing a pragmatic solution that requires collecting the resources suitable for paying for the expected risks as a collective.

28 Moreover, as we shall see next, the notion of individual risk proves elusive. If so, the principle of “individual responsibility for individual risk” seems hardly practically feasible, first, due to a hardly avoidable lack of societal agreement about how to measure responsibility in the general case, second, due to the elusive notion of individual risk, specifically.

29 The rejection of *separation* as a criterion of insurance fairness is coherent with FEC (Loi et al., 2019; Baumann & Heitz, 2022) assuming that the attribute J (that which justifies inequality) is *not* the actual economic value of the claims *actually* caused by the insured.

30 Notice that the metrics *outcome test* (or *hit rate*) and *click through rates*, which are often used in predictive policing and personalized online ads settings, respectively, are equivalent (Simoiu et al., 2017).

31 Here, we consider the weaker variant (*sufficiency* or *well-calibration*) for the fairness evaluation, even though the stronger one might make sense from an insurer to avoid an overall over- or underestimation of risks. In contrast, *sufficiency* as defined here allows an insurer to deviate from setting premiums that will equal the expected damages across all groups, e.g., to consider the willingness to pay of certain individuals, as long as this is done equally across the relevant groups *A*.

32 Notice that if there are more than two groups to consider, more sophisticated statistical techniques (such as ANCOVA) might be needed.

33 This is in line with the definition of group discrimination provided in Lippert-Rasmussen (2014), including condition (v), which implies, for indirect discrimination (where beliefs in the inferiority of certain groups and animosity are ruled out *a-priori*) that the practice is only discriminatory for people of one group if they are made worse off by the indirectly discriminatory practice in comparison to people of a different group.

34 In reality, in the bread case, a material good is sold, whereas, in the case of insurance premiums, it is an expectation that is sold.

35 Notice that additionally requiring the expected damages to be equal to the paid premium for all groups would be equivalent to *calibration within groups* (Kleinberg et al., 2016). *Sufficiency*, which is equivalent to *well-calibration* (Chouldechova, 2017) is a slightly weaker fairness criterion.

36 See Section 3.2 for a detailed explanation of why this is the case.

37 That is, socially salient groups according to the definition in Lippert-Rasmussen (2007).

38 See Section 4 for a practical example.

39 See Section 3.3.1 for a more detailed description of community ratings.

40 The use of gender has been forbidden for insurance underwriting in the European Union since the European Court of Justice’s so-called Test-Achat ruling in 2012 (Rebert & Van Hoyweghen, 2015).

41 In Section 4, we show how this definition of fairness can be practically assessed.

42 To preserve anonymity for the review process, the code will be made publicly available at a later stage.

43 See Noll et al. (2020), who provide a detailed description of the dataset.

44 GLMs are an extension of linear models that allow for non-normal dependent variables.

45 Here we briefly describe the columns used from the dataset *freMTPL2freq* for the practical example:

- ClaimNb: Number of claims during the exposure period.
- Exposure: The period of exposure for a policy in years.
- VehPower: The power of the car (ordered values).
- VehGas: The car gas, Diesel or regular.
- DrivAge: The driver age, in years (in France, people can drive a car at 18).
- Density: The logarithmic density of inhabitants (number of inhabitants per square-kilometer) of the city where the car driver lives in.
- Region: The policy region in France (based on the 1970–2015 classification).
- VehAge: The vehicle age, in years.

46 Data and Code used for this practical example are available at: https://github.com/joebaumann/fair-insurance-premiums.

47 Note that the claim frequency over all policyholders in the dataset (w.r.t. the exposure) is just over 10%. Hence, the vast majority of policyholders are claim-free in the accounting year. However, as the non-occurrence of such an event in a single year is not equivalent to a risk of 0, checking for the fairness definition *separation* (which requires that policyholders with equal claims pay equal premiums, on average) does not make any sense.

48 Similar to Lorentzen & Mayer (2020), both models used the *Exposure* as the weight and optimized the Poisson deviance, which measures how well the model fits the data.

49 Both Berk et al. (2017)’s and Steinberg et al. (2020a, 2020b)’s solutions are examples for a fairness mitigation techniques called in-processing. In the algorithmic fairness literature, many different approaches have been proposed to ensure fairness regarding a specific sensitive attribute, most of which can be classified into one of three mechanisms: pre-processing, in-processing, or post-processing. The goal of pre-processing is to eliminate direct and indirect discrimination by transforming the training data. Instead, in-processing wants to change the training phase to produce fair outcomes. Post-procession is applied after the modeling phase and treats the algorithm as a black box – optimal post-processing solutions are provided by Hardt et al. (2016); Corbett-Davies et al. (2017) for the fairness criteria *independence* and *separation*, and by Baumann et al. (2022) for the fairness criterion *sufficiency*. In this paper, we focus on defining a morally appropriate definition of fairness instead and point to Pessach & Shmueli (2020) and Caton & Haas (2020) for a more detailed description of how these different fairness mitigation mechanisms can be technically implemented.
